# Zinc Binding to S100B Affords Regulation of Trace Metal Homeostasis and Excitotoxicity in the Brain

**DOI:** 10.3389/fnmol.2017.00456

**Published:** 2018-01-17

**Authors:** Simone Hagmeyer, Joana S. Cristóvão, John J. E. Mulvihill, Tobias M. Boeckers, Cláudio M. Gomes, Andreas M. Grabrucker

**Affiliations:** ^1^WG Molecular Analysis of Synaptopathies, Department of Neurology, Neurocenter of Ulm University, Ulm, Germany; ^2^Cellular Neurobiology and Neuro-Nanotechnology Lab, Department of Biological Sciences, University of Limerick, Limerick, Ireland; ^3^Bernal Institute, University of Limerick, Limerick, Ireland; ^4^Biosystems and Integrative Sciences Institute, Faculdade de Ciências, Universidade de Lisboa, and Departamento de Química e Bioquímica, Faculdade de Ciências, Universidade de Lisboa, Lisboa, Portugal; ^5^Health Research Institute (HRI), University of Limerick, Limerick, Ireland; ^6^Institute for Anatomy and Cell Biology, Ulm University, Ulm, Germany

**Keywords:** Zn, zinc, S100B, DAMP, synapse, calcium, excitotoxicity, zinc sensor

## Abstract

Neuronal metal ions such as zinc are essential for brain function. In particular synaptic processes are tightly related to metal and protein homeostasis, for example through extracellular metal-binding proteins. One such protein is neuronal S100B, a calcium and zinc binding damage-associated molecular pattern (DAMP), whose chronic upregulation is associated with aging, Alzheimer’s disease (AD), motor neuron disease and traumatic brain injury (TBI). Despite gained insights on the structure of S100B, it remains unclear how its calcium and zinc binding properties regulate its function on cellular level. Here we report a novel role of S100B in trace metal homeostasis, in particular the regulation of zinc levels in the brain. Our results show that S100B at increased extracellular levels is not toxic, persists at high levels, and is taken up into neurons, as shown by cell culture and biochemical analysis. Combining protein bioimaging and zinc quantitation, along with a zinc-binding impaired S100B variant, we conclude that S100B effectively scavenges zinc ions through specific binding, resulting in a redistribution of the intracellular zinc pool. Our results indicate that scavenging of zinc by increased levels of S100B affects calcium levels *in vitro*. Thereby S100B is able to mediate the cross talk between calcium and zinc homeostasis. Further, we investigated a possible new neuro-protective role of S100B in excitotoxicity via its effects on calcium and zinc homeostasis. Exposure of cells to zinc-S100B but not the zinc-binding impaired S100B results in an inhibition of excitotoxicity. We conclude that in addition to its known functions, S100B acts as sensor and regulator of elevated zinc levels in the brain and this metal-buffering activity is tied to a neuroprotective role.

## Introduction

Metal ions play an important physiological role in synapse plasticity and function and thus their homeostasis is tightly regulated by transporters and metal-binding proteins. Recent evidence has revealed that several neuronal metalloproteins are candidates for prime regulators of metal homeostasis at the synapse (Bush, 2013; Cristóvão et al., 2016). In particular, trace elements such as iron, copper and zinc bind to these proteins, thereby influencing their conformation and functions (Leal et al., 2013). Disturbance of these processes leads to altered metal homeostasis and cellular distribution, which is a common feature of several neurodegenerative and neuropsychiatric diseases (Barnham and Bush, 2014).

S100 proteins are a family of regulatory, calcium-binding proteins that have been shown to have a tissue- and cell-specific expression and to act in a concentration-dependent manner (Donato et al., 2013). Calcium binding via EF-hand motifs induces conformational changes that underlie some of the functional interactions with other proteins. S100 proteins are also known to form functional and amyloidogenic assemblies (Yanamandra et al., 2009; Fritz et al., 2010; Carvalho et al., 2013, 2014).

S100B is mainly expressed in the central nervous system (CNS) where it accounts for 0.5% of soluble protein in the brain (Sorci et al., 1998; Donato and Heizmann, 2010; Ostendorp et al., 2011). At low concentrations (nM), S100B promotes neuronal survival (Villarreal et al., 2011) the induction of neurogenesis after traumatic brain injury (TBI) (Kleindienst et al., 2013), and the stimulation of neurite outgrowth (Huttunen et al., 2000; Villarreal et al., 2011; Saleh et al., 2013). Through activation and secretion at high concentrations (μM), S100B can also act extracellularly as signaling protein in an autocrine and paracrine manner (Sorci et al., 1998; Ponath et al., 2007; Villarreal et al., 2014). There, S100B acts via the RAGE (Receptor for Advanced Glycation End-Products) signaling pathway (Bianchi et al., 2011; Villarreal et al., 2014). RAGE activation increases extracellular S100 concentrations via NF-κB activation, which leads to a positive feedback cycle (Leclerc et al., 2010). Thus, neural S100 proteins are increased during processes activating NF-κB, such as inflammatory processes (Donato et al., 2013) and other insults to the brain. In particular, release of S100B was reported in response to excitotoxicity (Mazzone and Nistri, 2014).

*In vitro* studies have shown that S100B is an important factor in maintaining calcium homeostasis in astrocytes (Donato et al., 2009). Interestingly, besides calcium binding sites, various S100 proteins have additional regulatory binding sites for zinc and copper. S100B is able to bind two zinc ions per homodimer (Wilder et al., 2005; Charpentier et al., 2008) and by that the protein affinity for calcium increases (Baudier et al., 1986).

Zinc is one of the most prevalent trace metals in the brain and plays an important role as modulator of neurotransmission and signaling ion at synapses, thereby influencing processes such as synapse formation, maturation and plasticity. While most of the total brain zinc exists in a protein-bound state, “free” zinc ions can be found predominantly within synaptic vesicles (Palmiter et al., 1996; Cole et al., 1999), where zinc gets released together with glutamate from glutamatergic presynaptic terminals (Frederickson and Moncrieff, 1994; Frederickson and Bush, 2001). Alterations in brain zinc homeostasis have been reported associated with various disorders of the CNS. For example, abnormal levels of zinc have been implicated in neurodegenerative, neurological, and neuropsychological disorders such as mood disorders, autism spectrum disorders (ASD), Alzheimer’s disease (AD), Parkinson’s disease (PD), Huntington’s disease (HD), multiple sclerosis (MS), TBI and amyotrophic lateral sclerosis (ALS) (Pfaender and Grabrucker, 2014; Prakash et al., 2015). Intriguingly, an increase in S100B has been reported to occur in these disorders as well. Although excess of intracellular zinc is potentially neurotoxic, findings suggest that zinc acts protectively in situations of glutamate excitotoxicity via crosstalk to calcium signaling (Takeda, 2010).

It is well known that S100B binds and regulates protein targets as well as other calcium-signaling proteins in a calcium-dependent manner (Zimmer and Weber, 2010). Here, we investigate the zinc binding abilities of S100B dimer and tetramer and followed the question whether S100B may not only act as calcium but also zinc sensor, and whether zinc binding is necessary for physiological functions of S100B such as crosstalk with calcium signaling. Further, we followed the question whether high levels of S100B are able to affect local zinc concentrations by scavenging free zinc ions, which may lower toxic effects of zinc in situations of high zinc release such as over-excitation of glutamatergic neurons.

## Materials and Methods

### Materials

Zinpyr-1 was purchased from Sigma-Aldrich or Santa Cruz. Primary antibodies were purchased from Sigma-Aldrich (Map2, S100B), Invitrogen (tau), Synaptic Systems (S100B, Map2) and Origene (DDK). Secondary antibodies Alexa488, Alexa568 and Fluo4AM were purchased from Life Technologies. Secondary HRP antibodies were from DAKO. Unless otherwise indicated, all other chemicals were obtained from Sigma-Aldrich.

### Hippocampal Culture from Rat Brain

Pregnant rats were purchased from Janvier Labs. All animal experiments were performed in compliance with the guidelines for the welfare of experimental animals issued by the Federal Government of Germany and approved by the Regierungspraesidium Tuebingen and the local ethics committee at Ulm University (Ulm University, ID: O.103). The preparation of hippocampal cultures was performed essentially as described before (Grabrucker et al., 2009) from rat (embryonic day-18; E18). After preparation the hippocampal neurons were seeded on poly-l-lysine (0.1 mg/ml; Sigma-Aldrich) glass coverslips in a 24 well plate at a density of 3 × 10^4^ cells/well or 10 cm petri dish at a density of 2.5–3 × 10^6^ cells/dish. Cells were grown in Neurobasal™ medium (Life Technologies), complemented with B27 supplement (Life Technologies), 0.5 mM L-Glutamine (Life Technologies) and 100 U/ml penicillin/streptomycin (Life Technologies) and maintained at 37°C in 5% CO_2_.

### Cloning of Myc-DKK Tagged S100B and Mutagenesis of Zinc Binding Amino Acids

The pLenti S100B vector (Origene) was used for the mutagenesis of S100B zinc binding sites and carries a C-terminal Myc-DKK tag. Mutations were introduced at p.His15Ser, p.His25Ser, p.Cys84Ser and p.His85Ser through site directed mutagenesis (Agilent Technologies, QuikChange II XL Site-Directed Mutagenesis Kit) and this Myc-DKK tagged construct is thereafter referred to as S100B mut. Afterwards the Myc-DKK tagged S100B constructs (WT and zinc mutant) were cloned into a pGEMEX expression vector for protein production in *E. coli*.

### Recombinant Expression and Purification of S100B

Human wt S100B was expressed in *E. coli* and purified to homogeneity in dimeric and tetrameric forms using previously established protocols (Botelho et al., 2012a,b) either in non-tagged and Myc-DKK tagged versions. The Zn S100B mutant was purified with a strong anion exchange chromatography (HiPrep Q FF 16/10, GE Healthcare), followed by a gel filtration (HiLoad 16/600 Superdex 75, GE Healthcare) and finalized with other strong anion exchange chromatography (HiPrep Q FF 16/10, GE Healthcare). Demetallated forms of wt S100B and S100B mutant were generated by incubating the protein with 0.5 mM EDTA and 300 fold excess of DTT for 1 h at 37°C and followed by elution in a gel filtration (24 mL S75 Tricorn, GE Healthcare).

### Circular Dichroism (CD) Spectrometry

Circular Dichroism (CD) measurements were performed on a Jasco J-1500 spectropolarimeter equipped with a Peltier-controlled thermostated cell support. Far-UV CD spectra (200–260 nm) and thermal denaturation curves (at 1°C/min from 20 to 90°C) were recorded for Myc-DDK-tagged wt S100B and S100B mut at 0.1 mg/ml in 50 mM mTRIS pH 7.4.

### Zinc Binding 4-(2-pyridylazo)resorcinol (PAR) Assay

Binding of zinc to either Myc-DKK tagged variants of wt S100B and S100B mutant was assessed spectrophotometrically using the 4-(2-pyridylazo)resorcinol (PAR) assay (Hunt et al., 1985; Säbel et al., 2009). The absorbance for the ZnH_x_PAR_2_ complex was measured at 494 nm on a SpectrostarNano (BMG Labtech) with quartz cuvettes of 1 cm path length and complex concentration estimated using *ε* = 71500 M^−1^.cm^−1^ (Kocyła et al., 2015). Zn-S100B protein interaction was carried out as described in Bajor et al. (2016). In brief, 10 μM Myc-DDK-S100B wt or Myc-DDK-S100B mutant were titrated with 0–40 μM ZnSO_4_ in presence of 50 μM PAR. Control titrations were performed with 50 μM PAR in 50 mM TRIS pH 7.4. The absorption spectra were collected from 200 nm to 600 nm at 25°C. The relationship between the concentration of the zinc ion associated with the complex and the absorbance at 494 nm allow qualitative estimation of zinc affinity of S100B protein variants (Bajor et al., 2016). Relative zinc binding is given by 1+[(Abs(ZnH_x_PAR_2_•S100B) − Abs(ZnH_x_PAR_2_))/Abs(ZnH_x_PAR_2_)], where Abs(ZnH_x_PAR_2_•S100B) is the PAR absorbance at 494 nm in the presence of S100B.

### ANS Fluorescence Kinetics

Binding of calcium to S100B variants was assessed spectrofluorimetrically using real time 8-Anilino-1-naphthalenesulfonic acid (ANS) fluorescence emission at 495 nm on a Jasco FP-8200 spectrofluorimeter equipped with a Peltier-controlled thermostated cell support set for 25°C, upon excitation at 370 nm. Calcium binding assays were performed with 5 μM of S100B variants in 50 mM TRIS pH 7.4, then adding 10-fold of CaCl_2_ after 10 min. Two-fold of ANS was added at the beginning of the assay.

### Lentivirus Production

Lentivirus was produced by transfecting pLenti S100B and packaging plasmids into HEK293T cells (grown in DMEM + 10% fetal bovine serum) using Lenti-vpak Packaging Kit (Origene). In brief, transfections were conducted 2.5 × 10^6^ overnight plated HEK293T cells using 5 μg of pLenti S100B and 6 μg packaging plasmids DNA per 10 cm plate. Culture medium was changed after 18 h. After 2 and 3 days of transfection, the first and the second batch virus-containing medium was collected, combined, passed through a 0.45 μm filter to remove cell debris, and frozen at −80°C. The viral titer was determined by Lenti X qRT-PCR Titration Kit (clontech).

### Treatment of Hippocampal Cells

#### Treatment with S100B

For the toxicity profile of S100B dimer and S100B tetramer primary neurons were treated with either dimer or tetramer in concentration range between 100 nM and 30 μM at DIV10 for 24 h or with 30 μM for 24, 48 and 72 h. Saturation assays were performed with 30 μM of S100B dimer or tetramer and 60 μM of different metal ions including ZnCl_2_ and CaCl_2_, DIV10 for 24 h. S100B dimer and tetramer were incubated with metal ions 1 h on ice previous to neuronal exposure. For S100B uptake study neurons were incubated on ice or at 37°C for 5 min previous to 10 min exposure to 60 μM S100B dimer or tetramer.

#### Glutamate Excitotoxicity Assay

Primary hippocampal neurons were treated with 10 μM ZnCl_2_ for 24 h previous to excitotoxicity assay to ensure presynaptic vesicle loading with Zn. To induce glutamate-dependent excitotoxicity, cell culture medium of DIV14 hippocampal neurons was replaced by fresh medium and neurons were exposed to 10 μM L-Glutamic acid or 10 μM L-Glutamic acid in combination with 30 μM S100B wildtype or mutant protein at 37°C for 1 h. Alternatively, 30 μM S100B wildtype or mutant protein were pre-incubated with 60 μM ZnCl_2_ on ice for 1 h previous to neuronal exposure to saturate zinc binding sites. Afterwards medium was removed and cells were rinsed twice with HBSS.

### Immunocytochemistry

For immunofluorescence, the primary cultures were fixed with 4% paraformaldehyde (PFA)/4% sucrose/PBS at 4°C for 20 min and processed for immunohistochemistry. After washing 2 × 5 min with 1× PBS with 0.2% Triton X-100 at RT, blocking was performed with 10% FBS/1× PBS at RT for 1 h, followed by the primary antibody at RT for 2 h. After a 3 × 5 min washing-step with 1× PBS, incubation with the second antibody coupled to Alexa488, or Alexa568 followed at RT for 1 h. The cells were washed again in 1× PBS for 10 min and counterstained with DAPI for 5 min and washed with aqua bidest and mounted with Vecta Mount.

### Measurement of Trace Metal Concentrations

For fluorescent Zn-staining and Ca-staining of cultured neurons, growth medium was discarded and the cells were washed with PBS. Coverslips were incubated with a solution of 5 μM Zinpyr1 or 4 μM Fluo4AM, respectively, in PBS for 1 h at RT. To validate zinc-binding capacity of mutated S100B, infected primary neurons were incubated with 25 μM Zinquin ethyl ester (Sigma Aldrich) at RT for 1 h. Afterwards, coverslips were rinsed with PBS and neurons were fixed with 4% PFA/4% sucrose/PBS at 4°C for 20 min, counterstained with DAPI and finally mounted with Vecta Mount.

### Protein Biochemistry

Dot blot analysis was performed using a PVDF membrane wetted with 100% methanol. The membrane was incubated with transfer buffer for 2–3 min and protein lysate spotted on and incubated overnight. Subsequently, the membrane was washed 2× with TBST buffer 0.05% and blocked with TBS containing 5% non-fat dry milk for 30 min at RT on a shaker, followed by application of the primary antibody for 2 h at RT on shaker. After washing four times for 5 min each with TBST buffer 5%, incubation with secondary HRP antibodies was performed for 1 h at RT. Immunoreactivity was visualized using the SuperSignal detection system (Pierce, Upland, CA, USA) and blots imaged using a MicroChemi Imaging System from Biostep.

### Statistics

Statistical analysis was performed using Microsoft Excel and averages tested for significance using SPSS version 20. For comparisons, analysis of variance (ANOVA, one way or Welch’s) was performed followed by Bonferroni or Tukey *post hoc* tests for within group comparisons. For comparisons of two independent groups, student’s *t*-tests was used. Data are shown as mean ± SEM. Significance levels were set at *p* < 0.05 (<0.05*; <0.01**; <0.001***).

#### Fluorescent Measurement

Acquisition and evaluation of all images were performed under “blinded” conditions. Fluorescence images were obtained using an upright Axioscope microscope equipped with a Zeiss CCD camera (16 bits; 1280 × 1024 ppi) using Axiovision software (Zeiss) and ImageJ 1.51j.

## Results

### S100B at Increased Extracellular Levels Is Not Toxic, Persists at High Levels and Is Taken up into Neurons

S100B proteins have intra and extracellular roles and are found in a dynamic range of concentrations and oligomeric states (Sorci et al., 1998; Donato, 2001; Donato et al., 2009; Leclerc et al., 2009). While S100B occurs in cells mostly as homodimers, the presence of higher-order S100B multimers (tetramers, hexamers and octamers) has been clearly demonstrated in human brain extracts (Ostendorp et al., 2007). In particular, upon neuronal injury, S100 proteins are secreted actively from astrocytes and oligodendrocytes accumulating extracellularly at micromolar levels, mostly as oligomers (Donato et al., 2013). Thus, to determine effects modeling the full scope of extracellular S100B biology, we exposed primary rat hippocampal cell cultures to both S100B dimers and tetramers.

**Figure 1:**
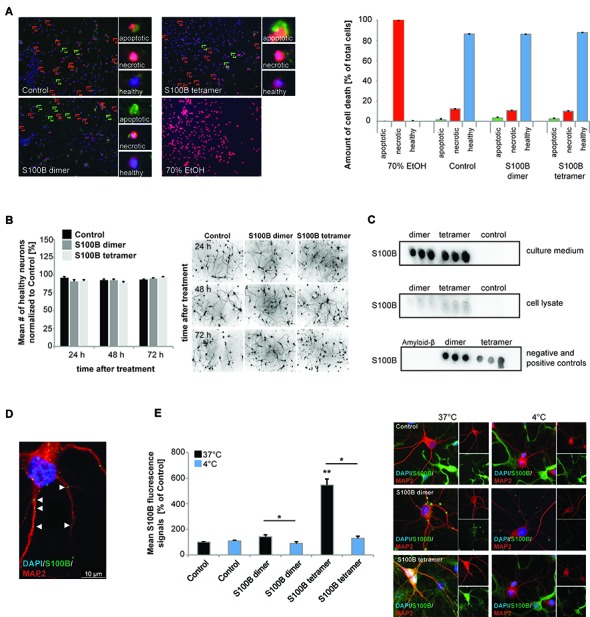
Exogenous application of S100B is non-toxic and leads to active uptake of the protein into cells. Treatment of hippocampal cultures with 30 μM dimeric and with 30 μM tetrameric S100B for 24 h at DIV10 was performed. **(A)** No significant differences in cell health were detected after application of 30 μM S100B dimer and tetramer. Apoptotic cells were identified using Annexin V—FITC (green signals), necrotic cells (red signals) by ethidium homodimer III and the total number of cells (blue signals) was assessed using Hoechst 33342 labeling all nuclei. A total of six optic fields of view per condition were analyzed. Ethanol treatment was used as positive control (Welch’s ANOVA, *F* = 312.579; *p* < 0.001; *Post hoc* analysis: control healthy cells vs. dimer healthy cells, *p* = 1.000; control healthy cells vs. tetramer healthy cells, *p* = 0.965; control healthy cells, dimer healthy cells, and tetramer healthy cells vs. 70% EtOH healthy cells, *p* < 0.001). **(B)** One time treatment of hippocampal neurons for 24 h, 48 h and 72 h also reveals no significant influence of S100B on cell health (one way ANOVA, *F* = 1.824; *p* = 0.085), assessed by DAPI staining of nuclei and visualization of dendrites by MAP2. Right panel: exemplary images showing MAP2 staining. **(C)** Dot-blot experiments show that after application of exogenous S100B dimer and tetramer, a high amount of proteins remains extracellular (upper panel). Weaker signals observed when cell lysates are used indicate that some of the protein is taken up into cells (middle panel). Medium and lysate of untreated cells were used as control. To confirm specificity of the antibody reaction, purified Amyloid-β, S100B dimer and tetramer were used (lower panel). **(D,E)** Hippocampal neurons show S100B immunoreactive puncta within their dendrites and cell soma. **(D)** Confocal fluorescent microscopy using *z*-stacks confirms the presence of intracellular exogenously applied S100B protein in vesicular like structures. **(E)** The S100B signal of neuronal soma increases significantly after application of exogenous S100B. This increase is abolished by interference with endocytotic processes by temperature reduction (Welch’s ANOVA, *F* = 31.823; *p* < 0.001; *Post hoc* analysis: control 37°C vs. S100B dimer 37°C, *p* = 0.061; S100B dimer 37°C vs. S100B dimer 4°C, *p* = 0.021; control 37°C vs. S100B tetramer 37°C, *p* = 0.004; S100B tetramer 37°C vs. S100B tetramer 4°C, *p* = 0.043). For quantification 10 cells per optic field and 5 optic fields per conditions were analyzed. Exemplary images are shown in the right panel. Neurons were visualzed using anti-MAP2 staining (red). Cell nuclei were labeled with DAPI (blue), and S100B (green) was labeled using anti-S100B antibody. MAP2 negative and S100B positive cells are astrocytes present in the cultures.

In a first set of experiments, we treated neuronal/glial cell co-cultures from rat with highly pure recombinant S100B for 24 h (Figure 1A; Supplementary Figures S1A–D). No significant impairments in cell health assessed by quantitative analysis of apoptotic and necrotic cells vs. healthy cells were observed after treatment at a physiological S100B concentration of up to 30 μM dimeric or 30 μM tetrameric S100B (Figure 1A), even if cells were exposed to S100B for up to 96 h (Figure 1B; Supplementary Figure S1E). Neuron morphology remained unaltered even after longer exposure to S100B dimers and tetramers, confirming that high levels of S100B do not affect neuronal health. Native gel and SDS-PAGE, as well as size-exclusion chromatography analyses showed that the 4ary structure of added S100B remained stable when added to cultures (data not shown).

Dot-blot experiments show that after application of exogenous S100B dimer and tetramer, a high amount of protein remains extracellular (Figure 1C). Noteworthy, S100B proteins supplied to the culture were also taken up into neurons (Figures 1C–E). Although glial cells have a much higher S100 signal intensity and astrocytes are the cell type expressing the majority of S100B *in vivo*, under control conditions, also neurons show S100B signals *in* *vitro* (Figure 1E). After application of S100B, S100B positive signals appear in vesicle-like structures in astrocytes as well as neurons (Figures 1D,E). These signals co-localize with Caveolin, but also Clathrin (data not shown). The concentration of S100B in cell lysates from hippocampal neurons increases after application of exogenous S100B dimer or tetramer (Figure 1C) and the level of intracellular immunoreactive S100B signals increases significantly in neurons (Figures 1D,E). This increase is abolished by blocking endocytotic mechanisms by shifting cells to 4°C (Figure 1E). S100B uptake has previously been described in astrocytes, differentiated SH-SY5Y cells and *Helix pomatia* neurons (Kubista et al., 1999; Yu and Fraser, 2001; Lasič et al., 2016).

From these results, we conclude that extracellular added S100B is not toxic to neurons and persists at high levels in cultures, in agreement with its known extra- and intracellular activities. Although in untreated control cells no S100B positive vesicle-like structures are visible in neurons, it cannot be excluded that even untreated neurons take up S100B in some situations.

### S100B Expressed in Neurons Leads to Sequestration and Intracellular Redistribution of Zinc

**Figure 2:**
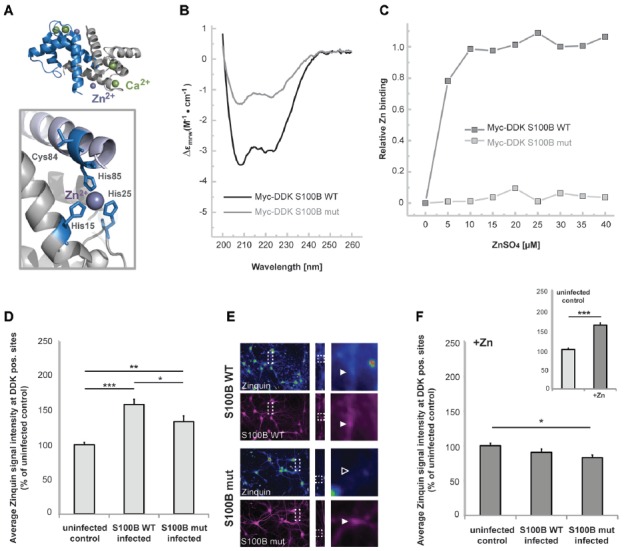
S100B sequesters zinc ions in hippocampal cells *in vitro*.** (A)** Illustration of the structure of S100B (PDB code 3d0y) showing the α-fold and zinc (purple sphere) and calcium (green spheres) binding sites; the enlargement represents the structural positions of residues involved in zinc interactions that were mutated to serines in the S100B mut variant. **(B)** Far-UV Circular Dichroism (CD) spectra of purified Myc-DDK-tagged wt S100B and mutant S100B at 0.1 mg.mL^−1^ in 50 mM TRIS-HCl pH 7.4, with typical α-helix minima observable at 208 and 222 nm. **(C)** Relative zinc binding to Myc-DDK tagged wt S100B (dark gray squares) is abolished in the Myc-DDK tagged S100B mutant (light gray squares), as inferred from PAR titration assays of S100B proteins (10 μM) with increasing ZnSO_4_ (at up to 40 μM) concentration. **(D)** Hippocampal neurons were infected at DIV1 with Lenti virus mediating expression of DDK-S100B wt, or DDK-S100B mut. The mean signal intensity of Zinquin fluorescence normalized to the co-localizing S100B fluorescence intensity is shown at DIV14. S100B is visualized by its anti-DDK tag. Cells expressing the mutant protein show significantly lower Zinquin signals at DDK-S100B mut positive sites compared to DDK-S100B WT positive puncta (one-way ANOVA, *F* = 19.347; *p* < 0.001; *Post hoc* analysis: control vs. S100B wt, *p* < 0.001; control vs. S100B mut, *p* = 0.004; S100B wt vs. S100B mut, *p* = 0.032). **(E)** Exemplary images showing infected cells. Zinquin signals are shown color-coded. Fluorescence intensities are assigned RGB colors. In S100B wt expressing neurons, a co-localizing Zinquin signal can be seen for S100B (upper panel). In cells expressing the mutated S100B, the fluorescence intensity of the Zinquin signal is significantly reduced (lower panel). S100B was visualized by anti-DDK antibody labeling (shown in magenta). **(F)** Addition of ZnCl_2_ has no effect on Zinquin signals associated with wild type S100B, and also does not lead to an increase in Zinquin signal associated with mutated S100B. Infected hippocampal cultures were treated with 60 μM ZnCl_2_ for 1 h. Addition of zinc significantly increases Zinquin fluorescence in control cells (insert; *t*-test, *p* < 0.0001). No further increase in Zinquin signal intensity was seen for wt S100B or mut S100B that showed significantly lower co-localizing Zinquin signal intensity (*n* = 10 cells; one-way ANOVA, *F* = 3.988; *p* = 0.03; *Post hoc* analysis: control vs. S100B mut, *p* = 0.024).

Considering that S100B is a calcium and zinc binding protein, we then tested the hypothesis that high extracellular levels of this protein influence the homeostasis of these neuronal trace metals. Thus, in the next set of experiments, we investigated whether elevated levels of S100B are sufficient to induce alterations in cellular zinc contents. To that end, we generated a mutant S100B protein variant (S100B mut), with Serine replacements of the amino acids involved in putative zinc binding (His15, His25, Cys84 and His85) (Wilder et al., 2003; Ostendorp et al., 2011), which are mostly located on S100B helices I and IV (Figure 2A). We used far-UV CD analysis to confirm that the Myc-DKK tagged S100B wt and S100B mut are expressed as folded proteins, retaining the S100-fold canonical α-topology (Figure 2B). Slightly decreased secondary structure content is observed in S100B mut, which is compatible with some structural loss caused by the four mutated residues in the protein, which nevertheless do not compromise the conformational stability, as both proteins exhibited comparable thermal denaturation profiles (data not shown). Impaired zinc binding by S100B mut, which was retained by the Myc-DDK tagged S100B wt was confirmed using the competition assay with the chromogenic Zn^2+^ ion chelator 4-(2-pyridylazo)resorcinol (PAR; Figure 2C).

We then expressed these Myc-DDK-tagged mut and wt S100B in neuronal cultures by Lenti-virus infection to analyze effects on cellular zinc pools, using uninfected cells as control. Interestingly, zinc ions and Zinquin signals were detected co-localizing with S100B, labeled by anti-DDK staining (Figures 2D–F). Zinquin ethyl ester is a cell-permeable, fluorescein-based probe that selectively detects free and weakly bound zinc. Hippocampal cell cultures expressing the wt protein show significantly increased Zinquin signals at DDK-S100B wt positive puncta compared to Zinquin signals within uninfected cells, further confirming the ability of S100B to sequester zinc ions. Hippocampal cells expressing the mutant protein show significantly lower Zinquin signals at DDK-S100B mut positive sites compared to DDK-S100B WT positive puncta, supporting the results of impaired zinc binding to mutated S100B (Figures 2D,E). Remaining zinc signals at DDK-S100B mut positive sites may be produced by recruitment of wildtype S100B expressed by cells in culture into complexes with mutated S100B or endocytotic vesicles. Exogenous addition of zinc by treatment with ZnCl_2_ significantly increased Zinquin fluorescence in control cells (Figure 2F insert), but did not lead to further enhancement of signals associated with wt S100B (Figure 2E), indicating that under physiological conditions, S100B is mostly zinc-bound. Again, significantly lower Zinquin signals were detected co-localizing with mutated S100B (Figure 2F).

**Figure 3:**
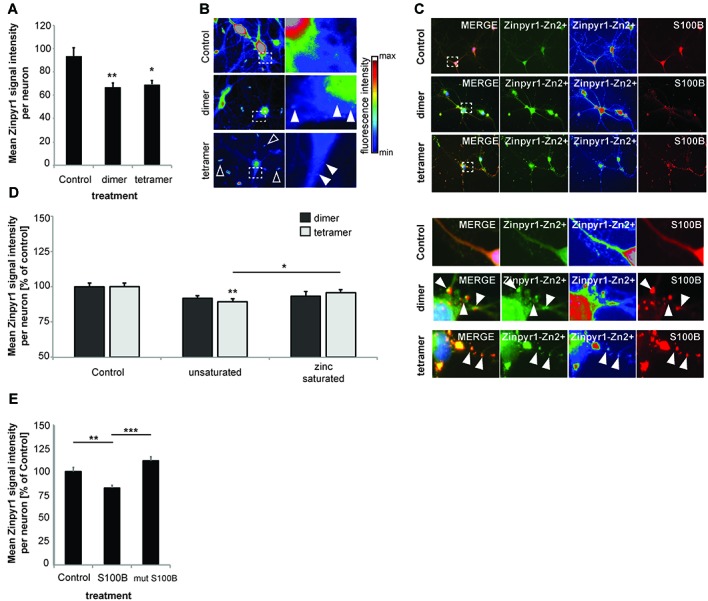
Increased levels of S100B sequester zinc ions. **(A)** Treatment of hippocampal cultures with 30 μM dimeric and with 30 μM tetrameric S100B for 24 h at DIV10 significantly reduces the intracellular Zn^2+^ concentration assessed by the analysis of Zinpyr1 signal intensity levels from 20 cells per condition for both application of the dimer and tetramer (Welch’s ANOVA, *F* = 8.084; *p* = 0.001; *Post hoc* analysis: control vs. dimer, *p* = 0.008; control vs. tetramer, *p* = 0.015). **(B)** Exemplary images showing signal intensities of Zinpyr1 in color—coded manner. Note the occurrence of Zn^2+^ containing clusters within neurons in cells treated with dimer and tetramer (full arrow) and extracellular Zn^2+^ positive accumulations (open arrow). **(C)** S100B signals co-localize with Zinpyr1 signals (full arrow) associated with Zn^2+^ after application of 30 μM dimeric or 30 μM tetrameric S100B for 24 h at DIV10. **(D)** Pre-incubation of S100B with 60 μM zinc leads to partial saturation of S100B zinc binding before application of 30 μM dimeric and with 30 μM tetrameric S100B for 24 h at DIV10, and thus to a significantly less decrease of intracellular zinc levels after treatment with S100B (Dimer: one-way ANOVA, *F*_(2,45)_ = 2.611; *p* = 0.085; Tetramer: *F*_(2,45)_ = 5.173; *p* = 0.01; *Post hoc* analysis: control vs. tetramer, *p* = 0.0045; tetramer vs. zinc saturated tetramer, *p* = 0.036; *n* = 16 cells). **(E)** Treatment of hippocampal cultures with 30 μM Myc-DDK-S100B wt, or Myc-DDK-S100B mut for 24 h at DIV10 leads to a significant decrease in intracellular Zinpyr1 fluorescence for wt S100B but not for the mutant S100B (one-way ANOVA, *F*_(2,77)_ = 8.86; *p* < 0.0001; *Post hoc* analysis: control vs. S100B, *p* = 0.0037; control vs. S100Bmut, *p* = 0.2469; S100B vs. S100Bmut, *p* < 0.0001).

Next, to investigate the consequences of the presence of increased amounts of S100B on global intracellular zinc and calcium levels, we again exposed primary hippocampal neuronal/glial cell co-cultures from rat to 30 μM dimeric or 30 μM tetrameric S100B for 24 h. We then assessed the intracellular zinc concentration by the analysis of Zinpyr1 signal intensity levels (Figure 3). Zinpyr1 is another cell-permeable, fluorescent probe that selectively detects free and weakly bound zinc, and that was used here because of its emission wavelength within the green spectrum. We found a significant decrease in intracellular zinc levels in cells exposed to both S100B dimer and tetramer (Figures 3A,B). Similar to the results obtained with Lenti virus based S100B expression, Zinpyr1 signals co-localize with S100B signals after exogenous application of the protein (Figure 3C).

Pre-incubation of S100B dimers or tetramers with zinc prior to application did not result in alterations in intracellular zinc levels. As referred above, application of dimer (as trend) and tetramer (significantly) reduced intracellular Zinpyr1 signal intensity. However, no such decrease was seen after application of the dimer or tetramer saturated with zinc (Figure 3D). Similarly, application of S100B mutant unable to bind zinc does not significantly reduce intracellular Zinpyr1 signal intensity (Figure 3E). Therefore, we conclude that S100B, either expressed in cells or added extracellular, effectively scavenges zinc ions through specific binding resulting in a redistribution of the intracellular zinc pool.

### S100B Mediates the Cross Talk between Calcium and Zinc Homeostasis: Increased Neuronal S100B Only Significantly Affects Calcium Levels upon Zinc Scavenging *in Vitro*

**Figure 4:**
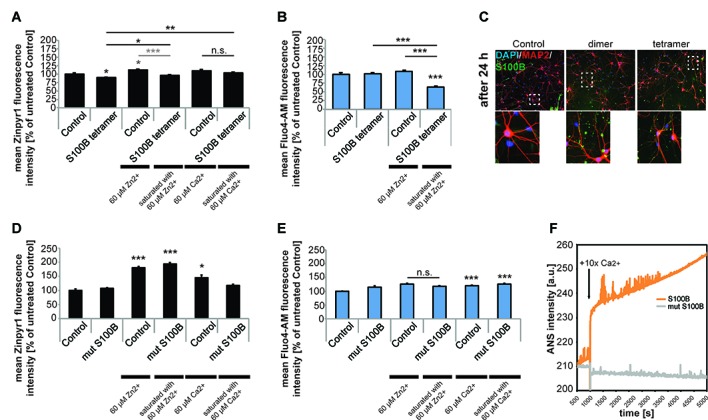
Only S100B saturated with zinc alters calcium levels in neurons *in vitro*. Hippocampal cultures were treated with 30 μM tetrameric S100B for 24 h at DIV10. **(A)** Application of S100B significantly decreases the intracellular zinc concentration measured by Zinpyr1 fluorescence intensity (*n* = 20 cells). Pre-incubation of S100B with 60 μM zinc leads to a significantly less decrease of intracellular zinc levels after treatment with S100B. Pre-incubation with 60 μM calcium also leads to significantly less reduction of intracellular zinc levels after S100B treatment (one-way ANOVA, *F* = 11.168; *p* < 0.001; Zn: *Post hoc* analysis: control vs. tetramer, *p* = 0.05; control vs. control + 60 μM Zn^2+^, *p* = 0.018; control vs. tetramer saturated with 60 μM Zn^2+^, *p* = 1.00; control + 60 μM Zn^2+^, vs. tetramer saturated with 60 μM Zn^2+^, *p* < 0.001; Ca: control + 60 μM Ca^2+^ vs. tetramer + 60 μM Ca^2+^, *p* = 0.573; tetramer vs. tetramer + 60 μM Ca^2+^, *p* = 0.006). **(B)** Application of S100B does not alter the intracellular calcium concentration measured by Fluo4-AM fluorescence intensity (*n* = 15 cells). Saturation of the zinc-binding site of S100B with zinc leads to a significant decrease in intracellular calcium as zinc binding increases the affinity for calcium binding in S100B (one-way ANOVA, *F* = 29.744; *p* < 0.001; *Post hoc* analysis: control vs. tetramer, *p* = 1.00; control vs. tetramer saturated with 60 μM Zn^2+^, *p* < 0.001, control + 60 μM Zn^2+^ vs. tetramer saturated with 60 μM Zn^2+^, *p* < 0.001; tetramer vs. tetramer saturated with 60 μM Zn^2+^, *p* < 0.001). **(C)** After treatment, S100B was visualized using ICC and cells counterstained with MAP2, and cell nuclei with DAPI. The staining reveals extracellular but also intracellular S100B in treated cultures associated with both, neurons and glial cells. **(D)** Hippocampal cultures were treated with 30 μM mutated dimeric S100B for 24 h at DIV10. Application of mutated S100B does not decrease the intracellular zinc concentration measured by Zinpyr1 fluorescence intensity (*n* = 20 cells). Pre-incubation of mutated S100B with 60 μM zinc leads to a significant increase of intracellular zinc levels after treatment, similar to the one seen after treatment of cultures with 60 μM zinc alone for 24 h. Pre-incubation with 60 μM calcium also leads to an increase of intracellular zinc levels (Welch’s ANOVA, *F* = 50.419; *p* < 0.001; *Post hoc* analysis: control vs. control + 60 μM Zn^2+^, *p* < 0.001; control vs. mut S100B saturated with 60 μM Zn^2+^, *p* < 0.001; control vs. control + 60 μM Ca^2+^, *p* = 0.011). **(E)** Hippocampal cultures were treated with 30 μM mutated dimeric S100B for 24 h at DIV10. Application of mutated S100B does not decrease the intracellular calcium concentration measured by Fluo4-AM fluorescence intensity (*n* = 20 cells). Pre-incubation of mutated S100B with 60 μM zinc does not lead to a decrease in intracellular calcium levels as after treatment seen for wt S100B. Pre-incubation with 60 μM calcium leads to a significant increase in intracellular calcium to a similar extent as incubation of cultures with calcium alone (Welch’s ANOVA, *F* = 9.475; *p* < 0.001; *Post hoc* analysis: control vs. mut S100B, *p* = 0.066; control + 60 μM Zn^2+^ vs. mut S100B saturated with 60 μM Zn^2+^, *p* = 0.64; control vs. control + 60 μM Ca^2+^, *p* < 0.001; control vs. mut S100B saturated with 60 μM Ca^2+^, *p* < 0.001). **(F)** Kinetics of ANS fluorescence upon addition of calcium to Myc-DDK-S100B wt and Myc-DDK-S100B mut shows that mutation of the zinc-binding site in S100B impacts the ability of S100B to bind calcium.

S100B protein binds both zinc and calcium ions and it is known that binding of zinc facilitates calcium binding. Thus, we next investigated whether not only zinc but also calcium levels are affected by the presence of increased levels of S100B. To that end, we applied S100B tetramer to primary hippocampal neurons. Application of S100B again significantly decreased the intracellular zinc concentration measured by Zinpyr1 fluorescence intensity. As seen before, pre-incubation of S100B with 60 μM zinc leads to a significantly less decrease of intracellular zinc levels after treatment with S100B (Figure 4A). Treatment of neuronal cultures with calcium alone slightly increases the intracellular zinc concentration (Figure 4A), possibly due to heightened pre- and post-synaptic zinc release following spontaneous neuronal activity (Grabrucker et al., 2014). Pre-incubation of S100B with 60 μM calcium leads to no significant decrease of intracellular zinc levels after S100B treatment. However, the zinc scavenging effect of S100B may be masked by unbound calcium present in the medium that slightly elevates zinc levels.

Calcium levels in turn remain unchanged after exposure of neurons to S100B tetramer (Figure 4B). However, binding of zinc increases the affinity of the S100B calcium sites. Thus, saturation of the zinc-binding site before application of S100B leads to a significant decrease in intracellular calcium compared to controls (Figure 4B). Visualization of S100B after application reveals extracellular but also intracellular S100B. Thus, the observed alterations in intracellular zinc and calcium levels might be caused by both extracellular S100B and intracellular S100B (Figure 4C). Application of mutated non-zinc binding S100B does not result in decreased intracellular zinc levels as seen before, and saturation of the mutated S100B protein does increase intracellular zinc levels to a similar amount as application of zinc alone to untreated cells. Thus, the increase is likely caused by unbound zinc in the peptide-containing medium. Again, application of calcium alone increased intracellular zinc levels, as does application of calcium saturated S100B, most likely due to the presence of unbound calcium (Figure 4D).

The zinc-dependency of the effect of S100B on calcium levels was further confirmed using the non-zinc binding S100B mutant. Application of mutated non-zinc binding S100B pre-incubated with zinc does not result in changes in intracellular calcium levels as observed for the wt S100B (Figure 4E), confirming that enhanced calcium binding by association of zinc with S100B is necessary to significantly affect intracellular calcium levels. Interestingly, the zinc-binding mutant of S100B has impaired calcium binding properties, which was evident from the purification, as binding of the calcium loaded S100B-mut to the phenyl sepharose-column was substantially diminished, indicating that the exposure of hydrophobic patches that takes place upon calcium binding is decreased. Calcium titration monitoring Anilinonaphthalene-l-sulfonate (ANS), a dye that binds to hydrophobic regions on the protein surface that in the case of S100B should be exposed after calcium binding, shows that the wt S100B binds calcium. However, calcium binding is almost absent in S100B with mutated zinc-binding site (Figure 4F). Calcium alone significantly increased intracellular calcium levels, as does application of calcium saturated S100B (Figure 4E).

Taken together, these results show that intracellular calcium levels of neurons are reacting to the local concentration of the S100B protein, and that alterations in S100B concentration alter the availability of calcium for neurons. Our results indicate that calcium-binding by S100B in neurons is significantly enhanced by the presence of high zinc concentrations which saturate the zinc-binding site of S100B. This crosstalk potentially affects multiple downstream synaptic processes but also patho-physiological events such as excitotoxicity. Intriguingly, S100B concentrations have been reported high under conditions facilitating excitotoxicity (Mazzone and Nistri, 2014).

### Zinc Binding to S100B Mediates Anti-excitotoxic Activity Effects

**Figure 5:**
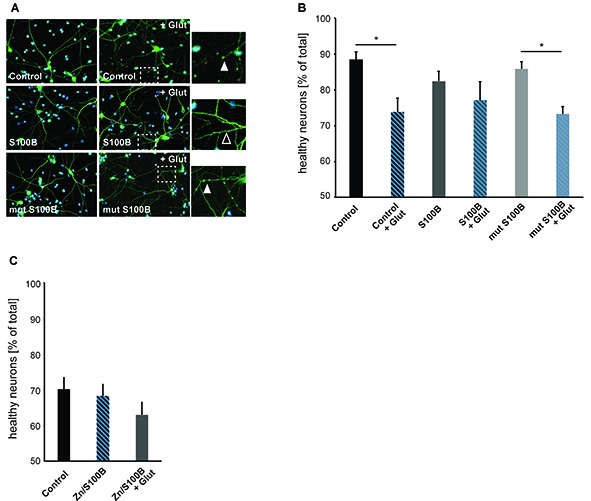
Zinc binding to S100B plays a physiological role in anti-excitotoxic activity. Hippocampal cultures were treated with 30 μM dimeric S100B or mutant S100B for 1 h at DIV14 with and without induction of excitotoxic effects by addition of 10 μM glutamic acid to the medium. **(A)** Neurons were visualized by DAPI (cyan) and anti-MAP2 (green) labeling. Fragmentation of dendrites as early sign of cellular toxicity is visible in control cultures after application of glutamic acid and after application of glutamic acid in presence of mutated S100B (full arrows). Co-application of wildtype S100B and glutamic acid does not induce significant dendritic fragmentation (open arrow). **(B)** The average number of healthy neurons assessed by DAPI signal and fragmentations of dendrites is significantly lower in control cultures treated with glutamic acid as a result of excitotoxicity. Co-application of S100B abolishes excitotoxic effects. Co-application of the zinc binding mutant S100B does not ameliorate excitotoxicity (one way ANOVA, *F* = 3.9226; *p* = 0.0042; *Post hoc* analysis: control vs. control + glut, *p* = 0.0329652; mut S100B vs. mut S100B + glut, *p* = 0.1169596; *n* = 10 optic fields of view per condition). **(C)** Hippocampal cultures were treated with 30 μM zinc-saturated S100B with and without induction of excitotoxic effects by addition of 10 μM glutamic acid to the medium at DIV14. The average number of healthy neurons assessed by DAPI signal and fragmentations of dendrites is not significantly different in cultures exposed to zinc-saturated S100B after induction of excitotoxicity (one way ANOVA, *F* = 1.2431; *p* = 0.3045; *n* = 10 optic fields of view per condition).

To investigate a possible new role of S100B as a neuro-protective mediator acting on excitotoxicity via its effects on calcium and zinc homeostasis, we induced excitotoxicity in hippocampal neurons *in vitro* using bath application of glutamate (Figure 5). We evaluated neuronal health by assessing the number of neurons showing fragmentation and beading of dendrites. Rapid, reversible changes in dendritic structure have been reported before under excitotoxic conditions (Park et al., 1996; Ahlgren et al., 2014). Consistent with findings of previous studies, beading of dendrites as early sign of cellular toxicity is significantly increased in control cultures after induction of excitotoxicity. Interestingly, in presence of S100B, excitotoxic effects are abolished. However, our data shows that this is only the case for S100B protein that retains zinc-binding capabilities, as non-zinc binding S100B mutant affords no neuroprotective action (Figures 5A,B). Reversibly, S100B saturated with zinc prior to exposure to glutamate is able to ameliorate more significantly the effects of excitotoxicity (Figure 5C).

Taken together, we conclude that the zinc binding capacity of S100B results in a physiological anti-excitotoxic function of the protein.

## Discussion

Synaptic biochemistry and function is tightly coupled to protein and metal ion homeostasis in the brain, where the levels of synaptic trace metals such as zinc, copper and iron, are regulated for example by changing the expression levels of metal binding proteins. This crosstalk is particularly evident in pathophysiological processes such as those occurring in protein deposition neurodegenerative diseases, which are also known to involve alterations in metal ion levels (Leal et al., 2013; Barnham and Bush, 2014; Cristóvão et al., 2016). For AD it has been shown that the aggregating amyloid β peptide binds zinc at physiological zinc levels, thus sequestering zinc ions and affecting synapse function (Adlard et al., 2010; Grabrucker et al., 2011).

In the brain, zinc is a potent neuromodulator. Upon synaptic activity, zinc is co-released with glutamate at “zincergic” synapses and is able to bind and modify postsynaptic receptors. Additionally, it may enter the postsynaptic compartment through ion channels. In case of over-excitation of neurons, for example resulting from seizures, stroke, or brain trauma, zinc translocation and zinc neurotoxicity may be a key component of excitotoxic effects (Frederickson et al., 1989, 2004). Both physiological and pathological transsynaptic movement of zinc may be modulated or sensed by extra- but also intra-cellular zinc binding peptides in the brain.

Among these zinc-binding proteins are some of the S100 family members, including S100B protein, which is very abundant in the brain and which binds both calcium and zinc. Genetic knockout of S100B results in viable and fertile mice that develop normally (Nishiyama et al., 2002a,b; Kim et al., 2011; Bluhm et al., 2015). However, studies revealed a role of S100B in hippocampal synaptic plasticity represented by its involvement in hippocampal-dependent learning and memory processes (Gerlai et al., 1995; Nishiyama et al., 2002a). Nevertheless, more than S100B depletion, elevated levels of S100B may occur under physiological and pathological conditions within the brain and have been implicated in several neurological disorders. The levels of S100B may rise to high concentration (μM) under some conditions and because of this, the concentration of S100B has been proposed as biomarker for pathological conditions of the brain, including perinatal brain distress, acute brain injury, brain tumors, neuroinflammatory and neurodegenerative disorders, and psychiatric disorders (Michetti et al., 2012).

However, so far, although some functions of S100B were reported to be zinc dependent, its activity has not been extensively linked to zinc homeostasis, although alterations in zinc might explain some of the reported functions of S100B.

Here, we made use of a hippocampal cell culture system to test the hypothesis that S100B plays a role in the regulation of neuronal zinc. Our results show that elevated concentrations of S100B indeed scavenge zinc ions in a magnitude that affects intracellular zinc levels *in vitro*. Given that measuring zinc has limited sensitivity using fluorophores, alterations in zinc levels need to be quite high. Therefore, we used S100B at high (μM), but non-toxic concentrations. Previously, μM S100B was reported to be neurotoxic *in vitro* (Huttunen et al., 2000; Vincent et al., 2007; Villarreal et al., 2011). However, the experimental conditions varied (experiments were not performed in rat primary hippocampal neurons, earlier time-points than DIV10 and DIV14 were used, and the treatment had different duration). In addition, studies with chick cerebral cortex neurons have shown that S100B at a μM concentration is beneficial for neuronal survival (Winningham-Major et al., 1989), and μM levels of S100B may occur under physiological conditions (Donato and Heizmann, 2010; Ostendorp et al., 2011).

We could show sequestration of zinc into S100B dimers and tetramers. It is likely that lower amounts of S100B will bind zinc ions and influence trace metal homeostasis as well. The S100B dimer coordinates at least two zinc ions (Wilder et al., 2003, 2005). Thus, the demand for zinc that usually occurs in low nM to pM range as free zinc in the extracellular space (Frederickson and Bush, 2001; Frederickson et al., 2006) as a result of zinc-buffering by proteins and ligands, and concentration in the range of pM in the cytoplasm of neurons, can be considered very high in case S100B reaches μM levels.

It is therefore likely, that S100B proteins are able to outcompete other zinc binding proteins with lower affinity for zinc. On the other hand, S100B may be able to lower elevated levels of zinc occurring under the same pathological conditions such as excitotoxic events, where S100B levels were reported high.

We showed that the effects of S100B on zinc homeostasis are direct effects resulting from zinc binding, as previous saturation of S100B zinc sites did not elicit the same alterations in neuronal zinc levels. In addition, we showed that extracellular S100B, at least in part, is endocytosed by neuronal cells. Thus, alterations in intracellular zinc may not only be caused by the generation of a gradient through increased extracellular concentrations of S100B, but also due to intracellular presence of the peptide.

**Figure 6:**
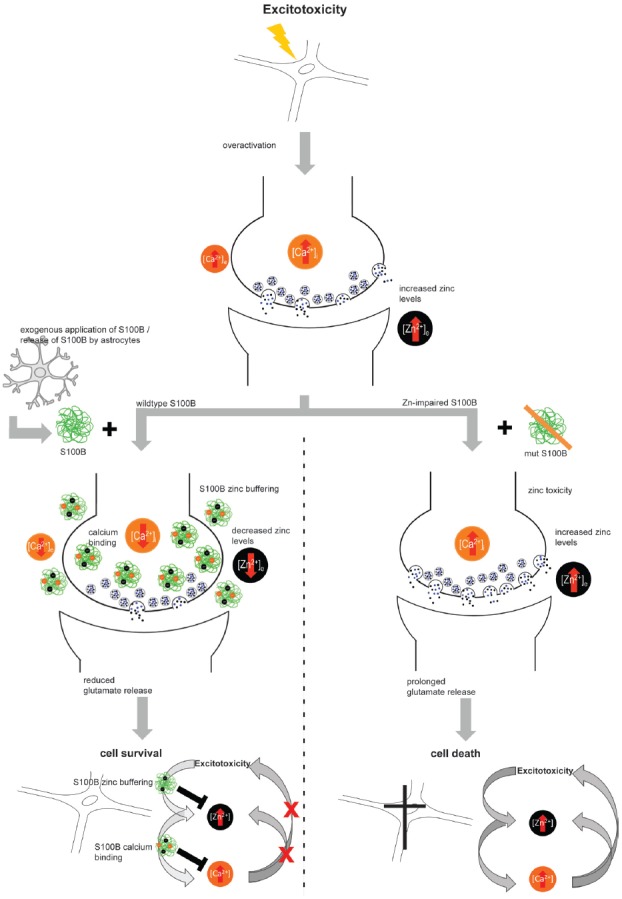
Hypothetical model for zinc-dependent anti-excitotoxic activity of S100B. In conditions of over-excitation, zinc is released from synapses in high amounts and calcium levels rise. Under these conditions, also S100B concentrations are found elevated. S100B may act as zinc buffering protein and upon zinc binding, the affinity for calcium binding is increased. Calcium binding of S100B may lower excitotoxic effects and thus promote neuronal survival. Excitotoxic calcium signaling was not altered in presence of S100B with mutated zinc binding sites and the effects on cell survival were abolished.

Finally, we could confirm that some S100B functions are indeed influenced by zinc binding and thus may be related to zinc sensing roles. We established that neuro-protective effects of S100B are zinc-dependent. Intriguingly, in conditions that favor excitotoxicity, not only zinc release may be increased but also S100B concentrations are often reported elevated. S100B may thereby act as zinc buffering protein. It was shown that zinc chelators are neuroprotectants in excitotoxicity (Frederickson et al., 2004) and chelators may mimic a role of S100B in these conditions. However, S100B may also be activated by zinc-sensing. Interestingly, zinc-binding to S100B mediates the ability of S100B to affect calcium levels. Mutation of the zinc-binding site impairs calcium binding to S100B. Intracellular calcium levels, and thereby possibly calcium signaling, were thus not altered in presence of S100B with mutated zinc binding sites, but intracellular calcium levels showed a decrease after application of zinc-bound S100B. It was shown before that zinc binding increases the affinity of S100B for calcium. In our experiments we could show that high levels of S100B, if saturated with zinc, may affect intracellular calcium homeostasis. Interestingly, it was shown that alteration of zinc homeostasis may modify glutamate excitotoxicity via crosstalk with calcium signaling (Takeda, 2010; Figure 6).

The effects of extracellular S100B on neurons have been reported to be mediated by RAGE so far and the activation of downstream signaling pathways such as ERK and NF-κB pathways. However, these pathways are also dependent on the availability of free zinc. For example, altered activation of ERK1/2 occurs under low zinc levels (Nuttall and Oteiza, 2012), and zinc has been suggested to be an important regulator of NF-κB (Kim et al., 2003). Thus, physiological responses to S100B may involve a crosstalk between the different pathways and trace metal homeostasis.

Based on our data, S100B knockout animals might be more susceptible to glutamate excitotoxicity. To our knowledge, this has not been investigated so far. However, it was shown that S100B overexpressing mice have a reduced excitatory postsynaptic response (Gerlai et al., 1995).

We conclude that while high concentrations of S100B dimers and tetramers are not toxic *per se*, they are able to induce changes in the levels of zinc. Abnormal accumulation of S100B under physiological zinc levels may thus result in local zinc deficiency. In AD, the presence of amyloid beta (Aβ) peptides sustains chronic activation of primed microglia resulting in increased levels of inflammatory factors. S100B proteins have gained attention in the pathological processes involved in AD (Wang et al., 2014), as altered levels of S100B have been shown to accelerate AD pathology (Mori et al., 2010). Depletion of zinc might be a contributing factor. In contrast, in conditions favoring zinc neurotoxicity, S100B acts neuro-protective by buffering zinc, and zinc-bound S100B may modulate excitotoxicity by altering calcium signaling.

## Author Contributions

SH carried out the experiments with JSC, wrote methods and revised the manuscript. JJEM and TMB contributed antibodies and reagents. CMG and AMG conceived of the study, participated in its design, coordination and data analysis. AMG drafted the manuscript with contribution from CMG and JJEM. All authors read and approved the final manuscript.

## Conflict of Interest Statement

The authors declare that the research was conducted in the absence of any commercial or financial relationships that could be construed as a potential conflict of interest.
