# Supplementary material for: Zinc Binding to S100B Affords Regulation of Trace Metal Homeostasis and Excitotoxicity in the Brain
Source: Front Mol Neurosci. 2018 Jan 17;10:456. doi: 10.3389/fnmol.2017.00456 (PMC5776125; doi:10.3389/fnmol.2017.00456)
Supplement: Supplementary file 1 [file Image_1.pdf]

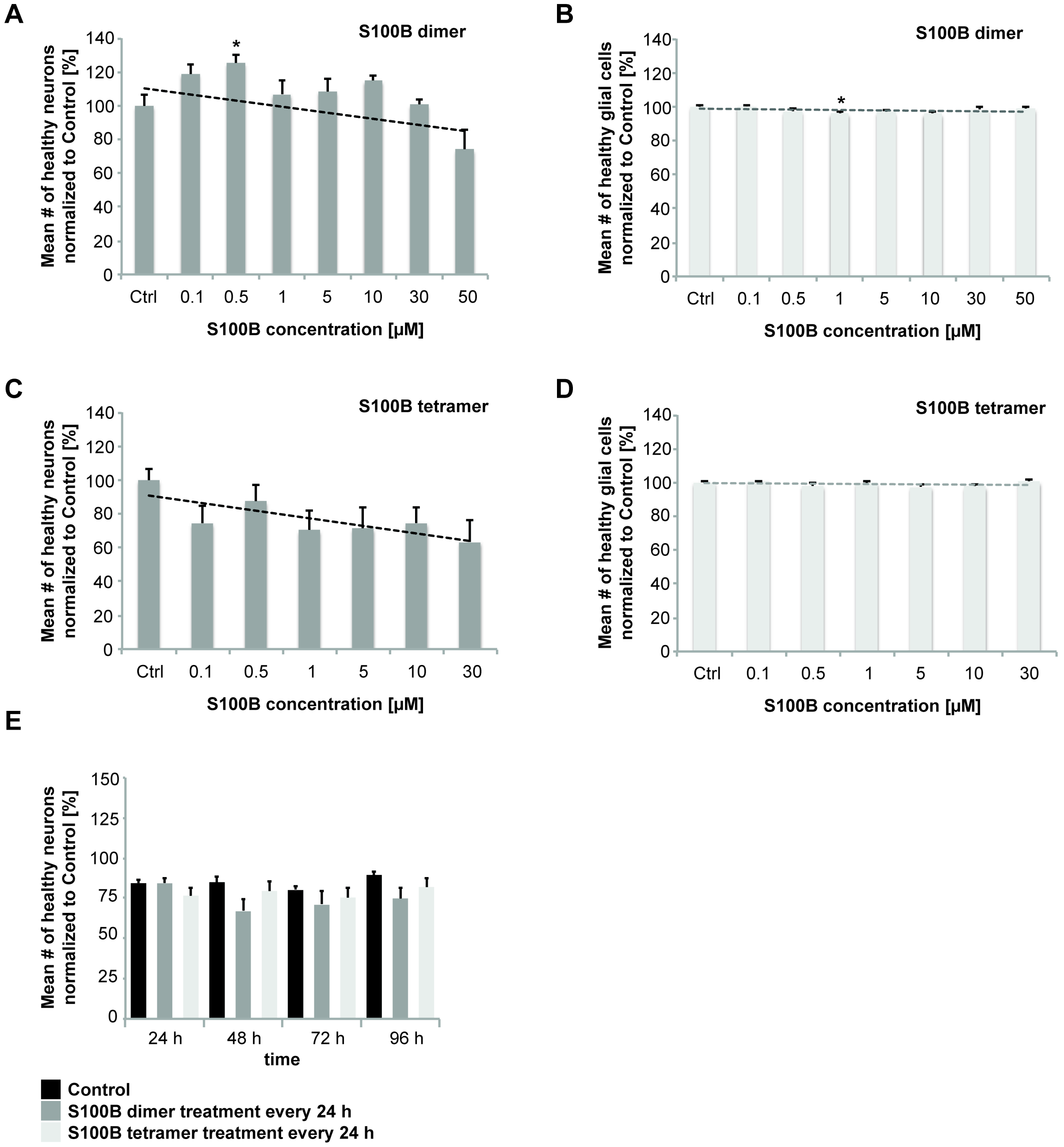

Figure S1: S100B dimer (A,B) and tetramer (C,D) were applied to neuronal (A,C) and glial cells (B,D) at DIV10 for 24 h. After the incubation time, cell health was assessed by counting cell density and apoptotic nuclei (identified by DAPI staining) from neurons (MAP2 positive cells) and glial cells from 10 optic fields of view per condition from two different preparations. The mean number of healthy cells is shown normalized to untreated control cells and the trend-line depicted. **(A)** Application of dimeric S100B peptides leads to increased cell health in nM concentrations and a trend towards slightly reduced cell health with increasing concentrations (Welch´s ANOVA, $F = 5.260$; $p < 0.001$; Post-hoc analysis: control vs. dimer 0.1 µM, $p = 0.144$; control vs. dimer 0.5 µM, $p = 0.017$; control vs. dimer 1 µM, $p = 0.978$; control vs. dimer 5 µM, $p = 0.943$; control vs. dimer 10 µM, $p = 0.183$; control vs. dimer 30 µM, $p = 0.998$; control vs. dimer 50 µM, $p = 0.617$). **(B)** Glial cells seem to be unaffected by equal concentrations of S100B (Welch´s ANOVA, $F = 2.576$; $p = 0.020$; Post-hoc analysis: control vs. dimer 1 µM, $p = 0.046$). **(C)** Similarly, treatment with

tetrameric S100B leads to a non-significant reduction in cell health at higher concentrations (one way ANOVA, $F = 1.235$; $p = 0.301$). **(D)** Again, glial cells were unaffected by tetrameric S100B in equal concentrations (Welch´s ANOVA, $F = 1.733$; $p = 0.130$). Given that 30 μM S100B dimer and tetramer did not show significant effects on the health, in further experiments, this concentration is used for the treatment of cells. **(E)** Treatment of hippocampal neurons with 30 μM S100B dimer or tetramer for 24 h, 48 h, 72 h, and 96 h with treatment every 24 hrs reveals no increase in cell death in treated cells compared to controls (Welch´s ANOVA, $F = 1.579$; $p = 0.080$). For all analyses, DAPI (to investigate apoptotic nuclei) and MAP2 staining (to visualize possible “pinching off” of dendrites) of 10 cells per condition and time-point were performed. Significances were tested using t-test.
